# Body Image Coping Strategies among Aesthetic Surgery Patients in Iran

**Published:** 2017-05

**Authors:** Rokhsareh Y. Yazdandoost, Niki Hayatbini, Mohammad Javad Fatemi

**Affiliations:** 1 Department of Plastic and Reconstructive Surgery, Burn Research Center, St. Fatima Hospital, School of Medicine, Iran University of Medical Sciences, Tehran, Iran;; 2 Department of Clinical Psychology, School of Behavioral Science and Mental Health, Iran University of Medical Sciences, Tehran, Iran

**Keywords:** Body Image coping strategies, Avoidance, Appearance fixing, Positive rational acceptance, Aesthetic surgery, Cosmetic surgery

## Abstract

**BACKGROUND:**

Aesthetic surgery procedures have been performed at dramatically increased rates in recent years in Iran. Few researches exist documenting the usage of body image coping strategies and its relationship in seeking surgery.

**METHODS:**

The present research examined data from 90 aesthetic surgery participants (30 Subjects each in invasive, minimally-invasive, and control groups). Assessed subjects on body image coping strategies inventory (avoidance, appearance fixing and positive rational acceptance) provided dysfunctional usage of its variables among Iranian clients.

**RESULTS:**

Between the three groups, on variables of body image coping strategies, there was a significant difference. There was a significant difference on avoidance variable in three groups. On positive rational acceptance variable, there was a significant difference for invasive group with minimally-invasive and control groups. No significant difference was found on appearance fixing variable.

**CONCLUSION:**

The study emphasizes on the role of psychological problems of aesthetic surgery clients that surgeons should be aware of them, which could inhibit the positive effects of aesthetic surgery. These results have implications for pre-surgical assessment along with psychological interventions at first step rather than invasive medical interventions.

## INTRODUCTION

Aesthetic surgeries have been increased at drastic rates all over the world in recentyears. In United States, ASPS members conducted a total of 1.7 million elective surgical procedures (such as breast augmentation, rhinoplasty, liposuction, and eyelid surgery) and 13.9 million minimally-invasive procedures (e.g., Botox injections, soft tissue fillers, chemical peels, etc.) during 2014. ASPS statistics revealed that breast augmentation decreased by 1%, eyelid surgery by 4%, face lift by 4%, and Botox injections increased by 6%, between the years of 2013 and 2014.^1^ Plastic surgeons observations in Iran indicated that the rate of plastic surgery among Iranians is increasing but the number of performed aesthetic procedures are underestimated due to numerous physicians from other specialties who offer aesthetic interventions.

Some researchers attribute these increase rates to negative body image because they believe that one’s physical appearance is based uponthe psychological structures of body image.^2^^,^^3^ Body image is a complex, multidimensional phenomenon consisting of one’s attitudes, perceptions, and experiences pertaining to one’s own physical appearance. Much contemporary body-image research derives from aperspective that conceptualizes body image as a function of cognitive social learning processes, including cognitive mediation of emotions and behaviors.^4^

The cognitive–behavioral model of Cashposits that both historical and proximal events shape and sustain body-image experiences. Historical influences are past events that predispose one’s thoughts and feelings about body image. These influences include cultural socialization concerning the values and standards of physical appearance, experiences in interactions and communication with others, one’s actual physical characteristics, and personality dimensions that affect body-image development.^5 ^Proximal processes entail events that trigger and maintain body-image experiences, which include self-dialogues and emotional inferences about the precipitant situation and the self. In turn, individuals engage in coping strategies to manage their potentially distressing body-image experiences. ^5^

Coping is a survival mechanism conceptualized as a transaction between an individual and the environment in which a response is directed at minimizing the psychological, emotional, and physical burdens associated with a stressful situation.^6^^,^^7^ Coping with body-image stressors in the context of a potential threat or challenge to body image, individuals develop and employ cognitive and behavioral strategies to adjust to or cope with these distressing thoughts, feelings, and situations.^5^ These strategies are maintained through negative reinforcement to the extent that they provide temporary relief through escape and avoidance, thus minimizing discomfort. Given the current literature, it is hypothesized that there is a significant difference in body image coping strategies between the three groups of invasive and minimally invasive aesthetic surgeries and control.

## MATERIALS AND METHODS

Research population included all clients who visited plastic surgeons private offices in Tehran seeking aesthetic surgery. The sample group consisted of 90 subjects (aged 25 to 45 years) in three groups of 30, the clients of invasive aesthetic surgery (including 21 volunteers of rhinoplasty and 9 volunteers of breast augmentation), with mean age of 32.5 years, minimally-invasive cosmetic surgery (10 volunteers of Botox injection, 13 volunteers of gel injection and soft tissue fillers injection, and 7 volunteers of microderm abrasion), with mean age of 34 years, and the control group, with mean age of 34.7 years. Selection of control group was based on cluster sampling method.

The sample group of invasive aesthetic surgery was chosen from those clients who had approached plastic surgeons’ offices in Tehran. The sample of minimally-invasive aesthetic surgery group was selected from those referred to one of the Tehran’s minimally-invasive aesthetic service provider centers. The control group Ss were randomly selected among employees from 4 departments (Medicine, Behavioral Science, Hygiene, Nursing and Midwifery) of Iran University of Medical Sciences. The control group was matched with aesthetic surgery groups based on age and education. The inclusion criteria were gender (female), age (25 to 45 years), education (minimum high school graduation), and having no history of any aesthetic surgery (ranging from minimally-invasive to invasive). 

A brief questionnaire concerning demographic information and current attitude and history of participants regarding aesthetic surgery was developed. Body Image Coping Strategies Inventory (BICSI) is an empirically validated assessment of cognitive and behavioral activities used to manage challenges to body image. It is comprisedof three individually scored subscales: avoidance, appearance fixing, and positive rational acceptance. The 8-item of Avoidance scale measured the extent to which an individual would avoid potential psychological discomfort through self-imposed ignorance of one’sundesirable thoughts or feelings. It determined good internal consistency, α=0.74. 

The 10-item of appearance fixing scale assessed how much energy the individual exerts trying to mediate poor body image with efforts to disguise, hide, camouflage, or alterthe body area that the individual deems undesirable, α=0.90–0.91. The 11-item of positive rational acceptance scale measured the individual’s more healthy approaches to divert attentionaway from the perceived flaw, which may include behavioralor mental strategies to pacify distress through endeavoring to perceivethe situation rationally as opposed to emotionally, α=0.80–0.85.^8^^,^^9^ At first, the purpose of conducting this research was explained to all the participants. Then, the clients were asked to read and sign a testimonial and answer the questionnaires. The data were analyzed through analysis of variance (F), followed by paired comparisons using SPSS software (Version 16, Chicago, IL, USA).

## RESULTS

As it was shown in Table 1, there were significant differences in 3 groups of this research study on the findings of body image coping strategies through BICSI namely, avoidance F (2,87)=19.724, *p*<0.001, appearance fixing F (2,87)=2.097, *p*<0.01, and positive rational acceptance F (2,87)=16.851, *p*<0.001. Table 2 shows significant differences (*p*<0.01), on paired comparisons between invasive aesthetic surgery group and minimally-invasive aesthetic surgery group on the 2 variables (avoidance and positive rational acceptance). Also, there were significant differences (*p*<0.01), on paired comparisons between invasive aesthetic surgery group and control group on the 2 variables (avoidance and positive rational acceptance), whereas there was no significant difference between minimally-invasive aesthetic surgery group and control group on positive rational acceptance variable, but between minimally-invasive aesthetic surgery group and control group, there was a significant difference (*p*<0.01), on avoidance variable. As far as, paired comparison was concerned, no significant differences between the groups on appearance fixing variable were found.

**Table 1:** Analysis of variance of Body Image Coping Strategies

**Variable**	**Sources of Change**	**SS**	**df**	**M** ^2^	**F**
Avoidance	Intergroup Intragroup Total	758.156 1672.067 2430.222	2 87 89	379.078 19.219	19.724****
Appearance fixing	Intergroup Intragroup Total	157.089 3258.733 3415.822	2 87 89	78.544 37.457	2.097**
Positive rational acceptance	Intergroup Intragroup Total	1514.467 3909.633 5424.100	2 87 89	757.233 44.938	16.851****

**** *p*<0.001,

** *p*<0.01

**Table 2:** Paired Comparisons of 3 groups on Body Image Coping Strategies variables

**Variable**	**Means differences**		
	**Invasive-minimally invasive**	**Invasive-control**	**Minimally invasive- control **
Avoidance	3.867**	7.100**	3.233**
Positive rational acceptance	-7.833**	-9.367**	1.533

** *p*<0.01

## DISCUSSION

The main purpose of this study was to compare the body image coping strategiesamong invasive and minimally-invasive aestheticsurgery applicants with control group on three variables namely avoidance, appearance fixing and positive rational acceptance through BICSI. Avoidance variable was a stronger predictor as compared to appearance fixing and positive rational acceptance in invasive aesthetic surgery clients, whereas positive rational acceptance variable was stronger for minimally-invasive aesthetic surgery clients and normal population.

Body image is a ‘‘multifaceted psychological experience of embodiment that encompasses evaluative thoughts, beliefs, feelings, and behaviors related to one’s own physical appearance’’.^10^ When triggered by contextual events, body image thoughts and emotions prompt adjustive, self-regulatory activities, or coping strategies such aspositive rational acceptance, avoidance, and appearance fixing.^9^ For those who have degrees of body image distress, desiring aesthetic surgery may be used as a coping strategy in order to eliminate intrusive or unwanted thoughts. This coping is problematic because it appears to be ineffective in reducing distress. Problematic coping strategies focusing onone’s appearance which results in avoidance strategies have been documented in previous studies.^8^^,^^9^

Study of coping strategies on participants’ considerations of elective aesthetic surgery demonstrated that participants with higher levels of body image disturbance who use avoidance and appearance fixing as a problematic coping strategy were significantly more likely to have considered elective aesthetic surgery.^11^ Whereas in present research, it is important to note that avoidance as a problematic coping strategy can predict seeking invasive aesthetic surgery. This may suggest that avoidance and considering seeking elective aesthetic surgery are both, ways of coping with body image disturbances, and that aesthetic surgery represents a more focus solution to that type of suffering by altering or eliminating that particular physical trait.^14^

While positive rational acceptance was more predictive among minimally-invasive aesthetic surgery clients and control group, focusing on one specific part of body may lead to desire in altering or eliminating that feature in an effort to reduce psychological distress through seeking surgery. Previous literature indicated that those who seek a medical route of coping will more often get what they request from plastic surgeons.^12^ The problem, of course, lies with those seeking surgery as a way of coping with aversive feelings of self-evaluation and distress because surgical interventions will not permanently solve these types of psychological problems,^13^ and may invite others.^14^

It may be important to develop screening methods for elective aesthetic surgery patients, more over considering them as invasive and minimally-invasive aesthetic surgery patients around problematic coping strategies such as avoidance, appearance fixing and positive rational acceptance to either stave off ineffective approaches to manage body image disturbance or at least educate potential surgery patients about the pitfalls of this strategy. Though there exist other reasons for seeking aesthetic surgery, those who do so as a means of coping with psychological distress may face more problems that lie ahead. Further research on problematic coping strategies with both clinical and non-clinical populations may help researchers to focus on psychological interventions addressing body image disturbance to help resolve this distress without undergoing unnecessary and perhaps problematic surgical interventions.^15^^,^^16^

Moreover, one of the psychological interventions that are more effective in reducing the use of avoidance and other problematic coping strategies, is cognitive behavior therapy. Accordingly, the basic concept of this therapy relies on the degree to which aesthetic surgery clients are invested in their appearance, depends greatly on the core self-schemas related to the appearance.^15^^,^^16^ These body image self-schemas serve as a cognitive template for one`s appearance evaluation and body image emotions. This type of intervention would emphasize on the dysfunctional thoughts and evaluations about the body and try to restructure these thoughts. This may help provide relief from distress and a way to more constructively engage in life. 

## Conflict of interest

The authors declare no conflict of interest.
